# Supplementary material for: Content-rich biological network constructed by mining PubMed abstracts
Source: BMC Bioinformatics. 2004 Oct 8;5:147. doi: 10.1186/1471-2105-5-147 (PMC528731; doi:10.1186/1471-2105-5-147)
Supplement: Additional File 2 — The original results of the above study (non-essential files are deleted to keep the file size under the limit set by BMC bioinformatics). [file 1471-2105-5-147-S2.bz2 › chilibotAdditionalFile2/dip30/728ID9822594E18078/html/TOLC_HLYD.html]

 


 **TOLC** and **HLYD** 
  
Found 21 abstracts in PubMed, retrieved 21.  
 

 What does Google say? 
 PDF only 
| .edu only 

---

**Interactive relationship** (e.g. stimulation, inhibition, etc)

**Neutral relationship**- Lipid bilayer experiments in vitro revealed specific interactions between  **TolC**  and vancomycin or  **HlyD**  protein.  Ref: 9393456 Mol Gen Genet, 1997
- These results are consistent with an interaction between  **HlyD**   **TolC**  and HlyB  **HlyD** .  Ref: 10071218 Mol Gen Genet, 1999
- A defect in  **TolC**  causes hyperresistance to vancomycin, even when present together with a mutant  **HlyD**  protein which in the presence of  **TolC**  renders E. coli hypersensitive to vancomycin.  Ref: 9393456 Mol Gen Genet, 1997
- The IM OM bridge was formed primarily by  **HlyD** , which assembled to stable IM trimers, corresponding to the OM trimers of  **TolC** .  Ref: 9822594 EMBO J, 1998
- This sequence of  **HlyD**  also seems to interact with a component of the hemolysin secretion machinery since a hybrid  **HlyD**  protein carrying the corresponding  **TolC**  sequence, although inactive in the transport of HlyA, is able to displace wild type  **HlyD**  from the secretion apparatus.  Ref: 7816028 Mol Gen Genet, 1994
- The second region of  **HlyD**  region B, located between amino acids Leu127 and Leu170, is highly homologous to the otherwise unrelated outer membrane protein  **TolC**   [ **TOLC** ] .  Ref: 7816028 Mol Gen Genet, 1994
- Two different mutations in  **hlyD**  alter the cell s susceptibility to vancomycin mutations in the  **tolC**  homologous region of  **hlyD**  increase vancomycin resistance.  Ref: 9393456 Mol Gen Genet, 1997

**Non-interactive relationship** (e.g. studied together, co-existance, homology, etc.)

- Moreover, they suggest that the HlyB  **HlyD**   **TolC**  based antigen delivery system with attenuated Salmonella spp.  Ref: 9119463 Infect Immun, 1997
- **HlyD**  and HlyB assembled a stable IM complex in the absence of  **TolC**  and substrate.  Ref: 9822594 EMBO J, 1998
- Our data suggest that all beta Gal HlyAs fusion proteins generated are secreted, albeit with varying efficiencies, by the HlyB  **HlyD**   **TolC**  hemolysin secretion machinery under Sec proficient conditions.  Ref: 8842146 Mol Gen Genet, 1996
- The system which consists of the two specific membrane proteins HlyB and  **HlyD**  and the outer membrane protein  **TolC**   [ **TOLC** ] , recognizes on HlyA a C terminally located signal sequence of about 60 amino acids.  Ref: 9382730 Behring Inst Mitt, 1997
- Our results are compatible with the  **HlyD**  cytosolic domain mediating transduction of the substrate binding signal directly, presumably to the  **HlyD**  periplasmic domain, to trigger recruitment of  **TolC**  and assemble the type I export complex.  Ref: 11676535 J Mol Biol, 2001
- Our data suggest that TnhlyAs generated hemolysin fusion proteins are secreted efficiently by the HlyB  **HlyD**   **TolC**  hemolysin secretion machinery.  Ref: 9711856 FEMS Microbiol Lett, 1998
- Secretion of Escherichia coli hemolysin is mediated by a sec independent pathway which requires the products of at least three genes, hlyB,  **hlyD**  and  **tolC** .  Ref: 7816028 Mol Gen Genet, 1994
- Together with the inner membrane protein  **HlyD**  and the outer membrane protein  **TolC**   [ **TOLC** ] , HlyB is responsible for transport of the 107 kDa toxin HlyA from the cytoplasm, across both membranes of the cell envelope of Escherichia coli, directly to the medium.  Ref: 8366127 J Biol Chem, 1993
- We show that an scFv HlyA hybrid secreted by Hly type I transporter  **TolC** , HlyB,  **HlyD**  is accumulated in the extracellular medium with the disulphide bonds correctly formed.  Ref: 11309117 Mol Microbiol, 2001
- The fragment contains four transcribed and translated genes inh, which codes for a protease inhibitor and is not required for protease secretion, and prtD, prtE and prtF, which share significant homology with the hlyB,  **hlyD**  and  **tolC**  genes required for alpha haemolysin secretion in Escherichia coli.  Ref: 2184029 EMBO J, 1990
- A recombinant r Salmonella typhimurium aroA vaccine that secretes the naturally secreted protein of Mycobacterium bovis strain BCG, Ag85B, by means of the HlyB  **HlyD**   **TolC**  export machinery termed p30 in the following was constructed.  Ref: 10727883 FEMS Immunol Med Microbiol, 2000
- The export of Escherichia coli hemolysin across the cytoplasmic and the outer membranes requires the COOH terminal signal sequence of HlyA, the two specific translocator proteins HlyB and  **HlyD** , and the outer membrane protein  **TolC**   [ **TOLC** ] .  Ref: 9630987 Nat Biotechnol, 1996
- **HlyD**  is normally highly stable but in the absence of  **TolC** , the steady state level of  **HlyD**  is greatly reduced and the protein has a half life at 37 degrees C of 36 min.  Ref: 10071218 Mol Gen Genet, 1999
- On the contrary, in the absence of both HlyB and  **TolC**  the half life of  **HlyD**  is approximately 110 min.  Ref: 10071218 Mol Gen Genet, 1999
- Extracellular secretion of HlyA by the TetC HlyB hybrid required  **HlyD**  and  **TolC** .  Ref: 1400227 J Bacteriol, 1992
- The structure and function of the components of the HlyA secretion apparatus, HlyB,  **HlyD**  and  **TolC** , have been studied in great detail.  Ref: 11755084 Trends Microbiol, 2002
- In contrast, when the charged amino acid residues were deleted, the substrate was still engaged by  **HlyD**  but engagement was unproductive, i.e.  **TolC**  recruitment was not triggered.  Ref: 11676535 J Mol Biol, 2001
- Our results are compatible with the idea that a  **TolC** , together with the  **TolC**  homologous part of  **HlyD** , forms a pore in the outer membrane through which hemolysin is released and vancomycin taken up.  Ref: 9393456 Mol Gen Genet, 1997
- This complex comprises a traffic ATPase HlyB and the 478 residue adaptor protein  **HlyD** , which contacts  **TolC**  during recruitment.  Ref: 11676535 J Mol Biol, 2001
- In this way, mutations in the known secretion genes, hlyB,  **hlyD**  and  **tolC** , were obtained.  Ref: 8437516 Mol Microbiol, 1993
- HlyA remained in the periplasm unless the  **hlyD**  and  **tolC**  gene products were present in addition to the hybrid.  Ref: 1400227 J Bacteriol, 1992
- A recombinant r Salmonella typhimurium aroA vaccine strain was constructed which secretes the naturally somatic protein of Listeria monocytogenes, superoxide dismutase SOD, by the HlyB  **HlyD**   **TolC**  export machinery.  Ref: 9119463 Infect Immun, 1997
- The secretion functions involved in alpha haemolysin secretion in E. coli hlyB,  **hlyD** ,  **tolC**  can also be used to complement alkaline protease secretion by E. coli, although less efficiently.  Ref: 1904127 Mol Microbiol, 1991
- The toxin HlyA is exported from Escherichia coli, without a periplasmic intermediate, by a type I system comprising an energized inner membrane IM translocase of two proteins,  **HlyD**  and the traffic ATPase HlyB, and the outer membrane OM porin like  **TolC** .  Ref: 9822594 EMBO J, 1998
- These data suggest that the HlyB  **HlyD**   **TolC**  based antigen delivery system with attenuated r S. typhimurium as carrier is capable of inducing an immune response against mycobacterial antigens.  Ref: 10727883 FEMS Immunol Med Microbiol, 2000
- This result shows that in the presence of HlyB removal of  **TolC**  renders  **HlyD**  more unstable than it is in the absence of both HlyB and  **TolC** .  Ref: 10071218 Mol Gen Genet, 1999
- In addition, we report on our r Salmonella typhimurium expression system combined with the HlyB  **HlyD**   **TolC**  export machinery for delivering the prominent mycobacterial antigen Ag85B for immune recognition.  Ref: 10076914 FEMS Immunol Med Microbiol, 1999
